# Design and performance of a poly(vinyl alcohol)/silk fibroin enzymatically crosslinked semi-interpenetrating hydrogel for a potential hydrophobic drug delivery

**DOI:** 10.1039/c9ra09344c

**Published:** 2019-12-12

**Authors:** Chunqing Niu, Xiang Li, Yiyu Wang, Xinyu Liu, Jian Shi, Xinyu Wang

**Affiliations:** Hubei Province Research Center of Engineering Technology for Utilization of Botanical Functional Ingredients, Hubei Engineering University Xiaogan 432000 People's Republic of China yiyiyuyu.7@163.com; Department of Machine Intelligence and Systems Engineering, Faculty of Systems Science and Technology, Akita Prefectural University Akita 015-0055 Japan; State Key Laboratory of Advanced Technology for Materials Synthesis and Processing, Wuhan University of Technology Wuhan 430070 People's Republic of China; Biomedical Materials and Engineering Research Center of Hubei Province, Wuhan University of Technology Wuhan 430070 People's Republic of China

## Abstract

In this study, in order to obtain hydrogels with good properties for sustained release of hydrophobic drugs or for tissue engineering, poly(vinyl alcohol) (PVA)/silk fibroin (SF) semi-interpenetrating (semi-IPN) hydrogels with varied ratios of PVA/SF were enzymatically cross-linked using horseradish peroxidase. A vial inversion test determined approximate gelation times of PVA/SF hydrogels ranging from 5 to 10 min. The hydrogels with varied ratios showed differences in pore size and morphology. Mass loss rate of hydrogels increased from 15% to 58% with increasing PVA concentration. Stable hydrogels with PVA/SF at 0.5 : 1 w/w showed the best swelling ratio values in distilled water (7.36). FTIR analysis revealed that silk fibroin in these hydrogels exhibited the coexistence of amorphous and silk I crystalline structures and the SF and PVA molecules interacted with each other well. The mechanical properties of the composite hydrogels were controlled by the SF content. From the cell viability results, it was found that the hydrogels exerted very low cytotoxicity. Paeonol was chosen as the hydrophobic drug model for release studies from the hydrogels. Paeonol can be uniformly loaded into the composite hydrogels using the emulsifying property of PVA and paeonol release from the hydrogels was dependent on the PVA/SF ratio. This study applied a novel type of enzymatically crosslinked semi-IPN hydrogel that may have potential applications in drug delivery.

## Abstract 2

Enzymatically cross-linked PVA/SF semi-IPN hydrogels with tunable pore structure have potential applications in sustained release of hydrophobic drug.

## Graphical abstract

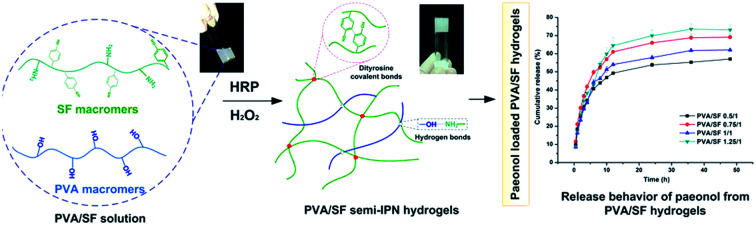

## Introduction

Hydrogels are three-dimensional networks composed of crosslinked hydrophilic polymers; they are water-insoluble while exhibiting a high degree of water uptake in aqueous environments.^1,2^ Hydrogels are particularly attractive for drug delivery and clinical application, as their properties can be fine tuned and they can encapsulate drugs, growth factors and other cell signaling factors to optimize treatment effect.^3,4^ A semi-interpenetrating hydrogel (semi-IPN) is a combination of polymers in a network form, in which one of the matrix constituents forms the network while the linear chains of the other constituent physically interact with each other and with the network.^5^ Combinations of different polymers have resulted in improved properties, like better elasticity and greater capacity to immobilize drugs.^6,7^ Due to this versatility, numerous synthetic and natural polymers and their combined hydrogels have been studied, such as polyurethanes, poly(vinyl alcohol), polyacrylamide, poly(lactic acid), sodium alginate, gelatin, collagen, silk fibroin (SF), *etc.*^6,8–14^

SF is a natural protein extracted from silk and widely used in the fields of biological materials and regenerative medicine due to its adequate sourcing, impressive mechanical properties, non-immunogenicity and good biocompatibility.^15–17^ SF solution can be obtained from refined silks and used to form different formats of biomaterial, including films, foams, scaffolds, mats and hydrogels.^18^ As a natural protein, SF can gel under certain conditions, including by physical and chemical methods. However, physical methods such as sonication, vortexing, surfactants and electric fields^19–22^ result in brittle behavior due to the formation of β-sheet structure. Chemical crosslinking methods can also be adopted to form gels, but introduce toxic chemical reagents that are difficult to remove.^23^ Recent reports indicated enzyme-catalyzed crosslinking of amino acid phenolic groups can be used to form hydrogels from natural polymers that contain the requisite phenol groups.^12,24,25^ Since tyrosine residues with phenolic groups account for more than 5% of the amino acids in silk, this approach was exploited as a crosslinking mechanism for fibroin gelation.

Although the SF hydrogel, with a microenvironment similar to the extracellular matrix, is often used as a hydrophilic drug carrier due to its high affinity to these drugs and its molecular permeability, it is difficult to load with hydrophobic drugs. SF can be blended or chemically crosslinked with other natural or synthetic polymers to improve the performance of single component hydrogels and expand their applications. In order to improve the drawbacks, poly(vinyl alcohol) (PVA) was chosen to interact with SF to generate a composite hydrogel with better properties for hydrophobic drug delivery. PVA is widely used in medical materials due to its good biocompatibility.^26^ Meanwhile, PVA is also an emulsifier, which can promote the uniform dispersion of hydrophobic drugs in water-based environments.^27^ Various researchers are working on the preparation methods and properties of SF/PVA composite materials. Lee *et al.* developed auricular cartilage using SF and PVA hydrogel based salt leaching, silicone mold casting, and freeze-thawing methods.^28^ Kundu *et al.* investigated photo-crosslinked SF/PVA hydrogels as a delivery system for macromolecular drugs.^29^ Li *et al.* prepared a SF/PVA porous scaffold *via* thermally induced phase separation (gelation) and freeze-drying process controlled delivery of curcumin.^30^

Paeonol is a typical hydrophobic drug which has attracted intense attention as a potential therapeutic agent against various cancers,^31^ but its application scope is limited by its water-insoluble property. In this paper, we demonstrated the preparation of PVA/SF semi-IPN hydrogel using HRP and H_2_O_2_. The prepared hydrogels were characterized with respect to morphology, stability, swelling, compressive mechanical properties, secondary structure and cytotoxicity. The release behavior of paeonol from the composite hydrogel was also evaluated.

## Experimental

### Materials

*Bombyx mori* raw silk fibers were purchased from Soho Biotechnology Co. Ltd. (China). PVA (MW ∼31 000, 88% hydrolysed), absolute ethanol, calcium chloride (CaCl_2_), sodium carbonate (Na_2_CO_3_), horseradish peroxidase (HRP), hydrogen peroxide (H_2_O_2_), and paeonol were purchased from Shanghai Aladdin Bio-Chem Technology Co., LTD (China). Cellulose dialysis membranes (molecular weight cut-off of 12 kDa) were purchased from Sigma-Aldrich (USA). Roswell Park Memorial Institute medium 1640 (RPMI-1640, Gibco), penicillin/streptomycin (HyClone), trypsin (HyClone) and fetal bovine serum (FBS, Gibco) were purchased from Shanghai Pufei Bio-Technology Co. Ltd. (China). Apoptosis and necrosis assay kit (Hoechst 33342 and PI), 3-(4,5-dimethyl-2-thiazolyl)-2,5-diphenyl-2-*H*-tetrazolium bromide (MTT), and dimethyl sulfoxide (DMSO) were purchased from Beyotime Institute of Biotechnology (China). L929 cell line (ATCC CCL-1) were purchased from American Type Culture Collection (ATCC, USA). All other chemicals were of analytical grade, purchased from Shanghai Sinopharm Chemical Reagent Co. Ltd. (China) and used without further purification.

### Preparation of silk fibroin solution

SF solution was prepared using a chemical degumming method before dissolution and dialysis. Raw silk fibers were treated three times in 0.05 wt% Na_2_CO_3_ solution at 98 ± 2 °C for 30 min to remove sericin. After being air-dried, the refined silks were dissolved in ternary solvent CaCl_2_ : CH_3_CH_2_OH : H_2_O (molar ratio = 1 : 2 : 8) with a bath ratio of 1 : 10 at 72 ± 2 °C for 1 h. Then, the mixed solution was dialyzed in deionized water for 4 days to get fibroin solution with a concentration of about 3 wt%. After dialysis, the solution was twice centrifuged at 8000 rpm for 20 min to remove silk aggregates and debris and then freeze-dried. SF solutions with different mass fractions were obtained by dissolving pure SF porous materials in deionized water. The SF concentration was determined gravimetrically after drying the SF solution at 60 °C.

### Preparation of PVA/SF semi-IPN hydrogels

Hydrogels were fabricated by the enzyme crosslinking method using HRP and H_2_O_2_ as in a previous report.^25^ Briefly, lyophilized HRP powder was mixed with sterile deionized water to form a stock solution with a concentration of 1000 U mL^−1^. PVA powder was added to distilled water and stirred at 60 °C until full dissolution to obtain PVA solution with different concentrations. 8% SF solution can be obtained by dissolving pure SF porous materials in deionized water. To produce PVA/SF hydrogels at weight ratios of 0.5/1, 0.75/1, 1/1, 1.25/1, 1.5/1 and 2/1, PVA (4–16% w/v) and SF (8% w/v) solutions were mixed together in equal volumes. After mixing thoroughly, the HRP solution was added to the mixed solution in a ratio of 10 Units of HRP to 1 mL of solution. To initiate gelation, 10 μL of 165 mM H_2_O_2_ solution was added per mL of solution for a final concentration of 1.65 mM and the solution was mixed by gentle pipetting prior to setting.

PVA/SF 1/1 hydrogels with paeonol concentrations of 1, 1.5 and 2 mg mL^−1^ were used for mass loss rate and swelling behavior tests and the final concentrations of paeonol in the semi-IPN hydrogel were 1, 1.5 and 2 mg mL^−1^, respectively. Briefly, 4 mg mL^−1^ of paeonol in 8% PVA solution was prepared by dissolving 400 mg of paeonol in 50 mL of 8% (w/v) PVA solution at 37 °C and stirring for 40 min to obtain homogeneity and then transferred to a 100 mL volumetric flask and brought to volume with 8% (w/v) PVA solution. This solution was thoroughly mixed with SF (8% w/v) solution at equal volume and finally enzymatically crosslinked by HRP and H_2_O_2_. The other two hydrogels with different paeonol concentrations were obtained using the same method but adjusting the paeonol concentration in the 8% PVA solution to 2 or 3 mg mL^−1^. In addition, PVA/SF 0.5/1, 0.75/1, 1/1 and 1.25/1 semi-IPN hydrogels with the same paeonol concentration of 1.5 mg mL^−1^ were used to test the hydrophobic release behavior. In this case, we prepared 3 mg mL^−1^ of paeonol in 4%, 6%, 8% and 10% PVA solutions and then mixed them separately with SF (8% w/v) solution at equal volume. The next steps were carried out using the above procedure to prepare these hydrogels.

### Gelation time

Gelation time was evaluated by a vial inversion test, where gelation time was designated as the time at which the solution no longer flowed after tilting the vial. Briefly, 10 μL of 165 mM H_2_O_2_ solution was added to PVA/SF solution containing HRP in an 8 mL scintillation vial and mixed with a pipette for 10 s, yielding a total of 1 mL of hydrogel solution. Samples were kept in a 37 °C water bath and gelation time was monitored. (*n* = 3).

### Scanning electron microscopy (SEM) and structure

The cross-sectional morphologies of the freeze dried PVA/SF hydrogels were examined using a JEOL JSM-5800 scanning electron microscope (SEM) at an operating voltage of 5 keV. Segments of cross-sectional surfaces were prepared by the slicing method and then sprayed with gold for SEM measurements. The pore sizes were determined by measuring 25 random pores from SEM images of the same sample using ImageJ software. The SEM images are recorded in BMP bitmap image format. The boundary of each hole in the surface layer of SEM images is determined according to the gradient method and an image of the surface layer of the cross section of porous materials is obtained. We calculate the area (*x*_1_, *x*_2…_*x*_*n*_) and the total area *S* (mm^2^) within the statistical region. Porosity (%) calculations used formula (1).^32^1
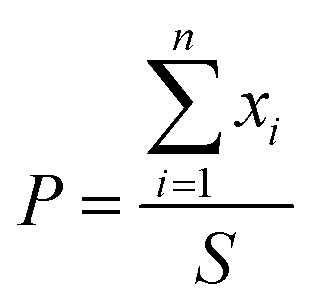


SF, PVA/SF and PVA/SF hydrogels containing paeonol were freeze-dried and powdered. The secondary structure was analyzed by Nicolet 6700 IR spectrometer (Thermo Fisher, USA). Fourier transform infrared (FTIR) spectra were obtained in the wave number range of 400 to 4000 cm^−1^.

### Mechanical performance

The compression performance of the sample was tested by a universal material testing machine at room temperature. The tests were carried out on an Instron 5967 universal mechanical testing machine (USA) equipped with a 50 N load cell at room temperature. The hydrogel samples to be tested were prepared into cylindrical shapes with a height of 10 mm and a diameter of 8 mm. For the compression test, the sample was compressed to ∼60% of the original height with a constant crosshead speed of 1 mm min^−1^. The stress–strain curves were drawn according to the data points and the average compression strength and compression modulus were calculated from the curves. Compression modulus (*E*) is defined as the slope of the initial linear portion of the stress–strain curve and compression strength is defined as the corresponding stress at 30% strain.

### Swelling and mass loss studies

The hydrogels were freeze-dried and their initial dry masses *m*_0_ were obtained. Then, the samples were immersed in deionized water at 37 °C for 24 h. The weights of the infiltrated hydrogels *m*_*t*_ were measured at regular intervals and the excess water on the surface was wiped off with filter paper. The swelling rate (SR) of the sample was calculated according to formula (2).2
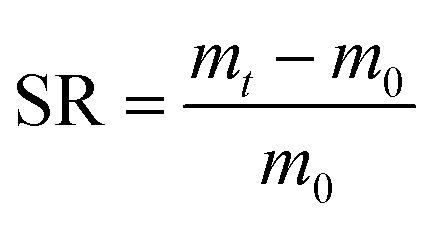


After freeze-drying, the hydrogels were dried at 60 °C to a constant weight, recorded as *m*_1_. The samples were placed into a centrifuge tube and deionized water was added at a bath ratio of 1 : 100. Samples were kept in an oscillation incubator for 24 h at 37 °C at 110 rpm. The remaining hydrogel was dried to a constant weight at 60 °C and reweighed; these values were recorded as *m*_2_. The mass loss rate (ML) of different hydrogels in hot water was calculated according to formula (3).3
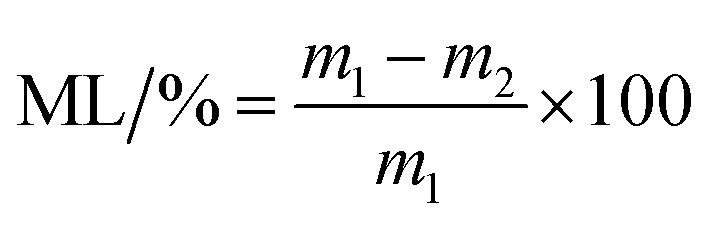


### Release of paeonol from PVA/SF semi-IPN hydrogels

Paeonol-loaded PVA/SF 0.5/1, 0.75/1, 1/1 and 1.25/1 semi-IPN hydrogels containing 1.5 mg mL^−1^ paeonol were prepared. Paeonol-loaded PVA/SF gels were incubated in 15 mL of PBS (pH 7.4) at 37 °C in an oscillation incubator (shaking speed 110 rpm) for sustained release testing. At different time points, 3 mL of sample buffer were taken and replaced by the same volume of fresh PBS buffer. The buffer samples collected at different times were stored at −20 °C for further analysis. The paeonol concentration was measured at 274 nm using a Persee T9S ultraviolet spectrophotometer (China). The released amount of paeonol was calculated by referring to the standard curve of paeonol. The drug release amounts were normalized to the drug amounts initially loaded on the SF/PVA hydrogels.

### Cytotoxicity test

To investigate the cytotoxicity of PVA/SA hydrogels with different ratios, an extraction process was performed according to the ISO 10993-5 standard method. The lyophilized hydrogels (3 mm thickness) were sterilized by immersion in 75% ethanol for 30 min, then hydrated and thoroughly rinsed with PBS. The conditioned media were obtained by incubating the hydrogels in 1 mL RPMI-1640 medium with a surface area of 3.5 ± 0.5 cm^2^ at 37 °C for 24 h. A similar amount of the culture medium was kept in the same conditions to be used as a control. L929 cells were incubated in RPMI-1640 medium supplemented with 10% FBS and 1% streptomycin–penicillin in a humidified atmosphere of 5% CO_2_ at 37 °C for 24 h. Cells were seeded at a density of 1 × 10^4^ cells per well on 96-well tissue culture polystyrene plates the day before experiments and then incubated with extracts of different hydrogels for 1,3 and 5 days. A similar amount of the culture medium was kept in the same conditions to be used as a negative control. A 10% solution of DMSO prepared in fresh culture medium was used as a toxicity positive control. At each defined time point, cell viability was assessed by MTT according to the manufacturer's instructions and the absorbance was measured at 570 nm using an EnSpire microplate reader (PerkinElmer, Singapore). Cytotoxicity was expressed as the percentage of cell viability relative to the negative control. The cells were stained with Hoechst 33342 and PI fluorescent dyes on day 3 and then examined by fluorescence microscope (Olympus, IX71, Japan).

## Results and discussion

### Preparation of semi-IPN hydrogels and gelation time

**Fig. 1:**
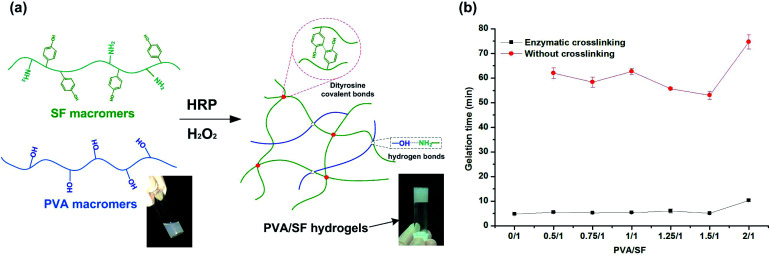
(a) A schematic diagram representing the formation of PVA/SF hydrogels. (b) Gelation times of PVA/SF hydrogels with different PVA/SF ratios.

Different fabrication strategies are utilized for hydrogel fabrication, among which the enzymatic cross-linking method is attractive since it allows hydrogels to be generated *in situ* with mild conditions and minimal cytotoxicity.^12^Fig. 1(a) is a schematic diagram of the formation of PVA/SF hydrogel using HRP and H_2_O_2_. Formation of phenolic radicals between SF macromers leads to polymerization of the precursor hydrogel solutions with HRP and H_2_O_2_, which play a key role in gel formation. In this reaction, HRP catalyzes a reaction generating tyrosine radicals in SF macromers which can then react with one another to form dityrosine covalent bonds between SF chains. Other intermolecular interactions, mainly due to hydrogen bonding by the hydroxyl groups of the PVA-amide groups of SF, occur between the two polymers, increasing the strength of the PVA/SF hydrogel network.^30^

Gelation times of the PVA/SF hydrogels ranged from approximately 5 to 10 min, as determined by a vial inversion test. Pure PVA solution cannot form a gel within 24 h by enzymatic crosslinking due to its lack of reactive groups. Gelation times of PVA/SF hydrogels at different ratios are shown in Fig. 1(b). After enzymatic crosslinking, the gelation times were significantly shortened compared with those without HRP. The gelation time of pure SF was the shortest, taking only 4.8 min. When the PVA/SF ratio was less than 1.5/1, PVA did not exhibit much effect on the gelation time, but when PVA/SF increased to 2/1, the gelation time significantly extended to 10 min. This may be due to the increase of PVA content affecting, to a certain extent, the homogenization of the solution and reducing the collision probability of reactive groups in SF macromolecules, thus extending the reaction time. As relatively short gelation time and mild conditions are used, biological molecules like growth factors and drug molecules can be encapsulated and loaded without losing their activity.

### Morphology of PVA/SF semi-IPN hydrogels

**Fig. 2:**
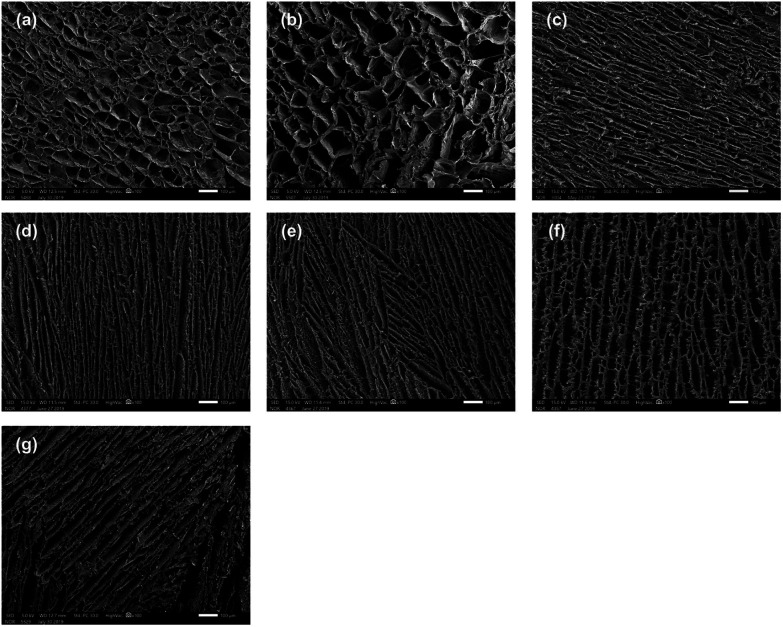
SEM images of PVA/SF hydrogels with different ratios: (a) 0.5/1, (b) 0.75/1, (c) 1/1, (d) 1.25/1, (e) 1.5/1, and (f) 2/1; (g) SEM image of PVA/SF 1/1 hydrogel without enzymatic crosslinking. Scale bar = 100 μm.

**Fig. 3:**
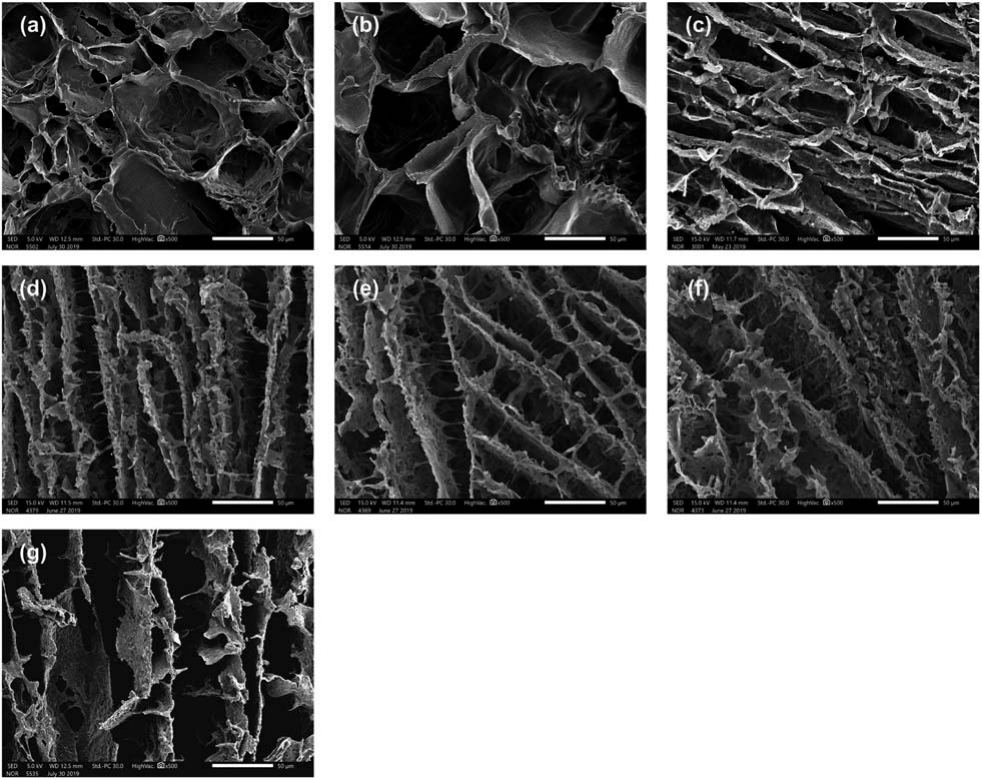
Enlarged SEM images of PVA/SF hydrogels with different ratios: (a) 0.5/1, (b) 0.75/1, (c) 1/1, (d) 1.25/1, (e) 1.5/1, and (f) 2/1; (g) enlarged SEM image of PVA/SF 1/1 hydrogel without enzymatic crosslinking. Scale bar = 50 μm.

The morphology of the cross sections of the PVA/SF semi-IPN hydrogels was investigated by SEM. SEM images of different hydrogels are shown in Fig. 2 and 3, demonstrating that all the samples with different ratios of PVA and SF had different three-dimensional porous structures. As seen in the enlarged SEM images, the hydrogels exhibited homogeneous distribution and interconnected porous structure, with no significant phase separation observed. The porosities and pore diameters of the PVA/SF hydrogels with different ratios are shown in Table 1.

**Table 1:** Porosity and pore size of PVA/SF hydrogels with different ratios

Sample	PVA/SF 0.5/1	PVA/SF 0.75/1	PVA/SF 1/1	PVA/SF 1.25/1	PVA/SF 1.5/1	PVA/SF 2/1	PVA/SF 1/1 without crosslinking
Porosity (%)	89.59	87.54	82.03	77.11	79.92	83.12	80.36
Pore size (μm)	47.75	67.28	32.19	37.02	39.67	45.49	43.21

As shown in Fig. 2(a)–(f), the pore structure changed depending on the blend ratio. When the proportion of PVA was small, as when PVA *versus* SF was 0.5/1 and 0.75/1, the hydrogels exhibited uniform oval-shaped pore structure. However, when PVA/SF increased to 1/1, uniform lamellar structure began to appear, the density of the hydrogel network increased dramatically, and the pore diameter and porosity decreased significantly to 32.19 μm and 82.03% from 67.28 μm and 85.54%, respectively. The pore diameter seemed to reach the minimum value in PVA/SF 1/1. When PVA content continued to increase, lamellar structure became more obvious and the pore structure became longer and thinner. Also, many nanofibers appeared among pore walls (Fig. 3(d)–(f)) and the pore walls became rougher. Fig. 2(g) and 3(g) showed that PVA/SF 1/1 hydrogel made without adding HRP and H_2_O_2_ exhibited less uniform distribution and the internal pores showed looser pore structure compared to crosslinked hydrogels. From the SEM images, the samples with SF/PVA ratios from 0.5 : 1 to 2/1 displayed variable pore structure and density, which can be used to control the diffusion of drug molecules and regulate the release behavior of the drugs.

### Mass loss rate and swelling behavior

**Fig. 4:**
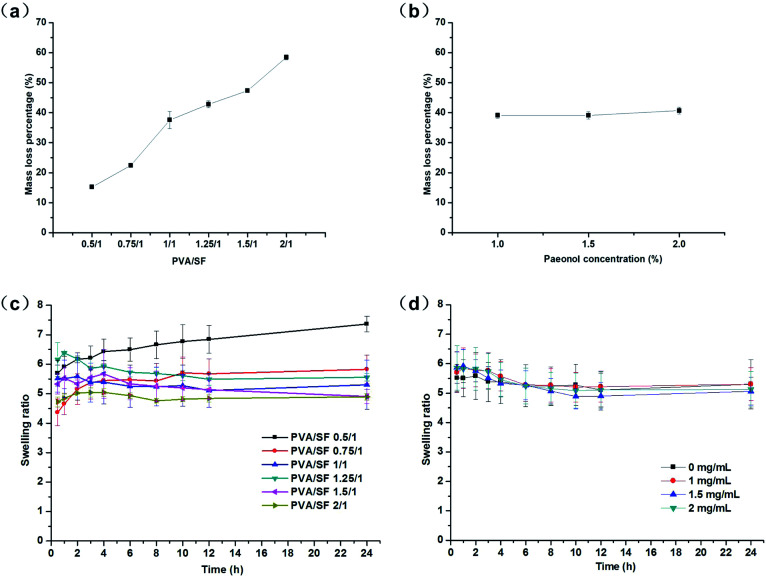
(a) Mass loss rates of PVA/SF hydrogels in water at 37 °C. (b) Mass loss rates of paeonol-loaded PVA/SF hydrogels in water at 37 °C. (c) Swelling ratios of PVA/SF hydrogels in water at 37 °C against time as a function of composition. (d) Swelling ratios of paeonol-loaded PVA/SF 1/1 hydrogels in water at 37 °C against time as a function of paeonol concentration.

As shown in Fig. 4(a), the mass loss rate of pure fibroin hydrogel was 12.63%, while the mass loss rates of PVA/SF hydrogels depended on the blend ratio of PVA/SF. As the PVA content in the gels increased, the mass loss rates of the hydrogels in hot water also increased. When PVA *versus* SF was 0.5/1, the mass loss rate of hydrogel was only 15.24%, as low as that of the pure fibroin hydrogel made using the same method, and the mass loss rates continued to rise steadily as PVA content increased; finally the value of mass loss rate in hot water reached the maximum at 58.45%. This may be because, in the PVA/SF hydrogel network, the enzymatically cross-linked fibroin macromolecules are the key skeleton structure, while the PVA chains physically interact with SF through weak molecular forces-hydrogen bonding. In aqueous solution, due to the hydration between PVA and H_2_O, the PVA chains with weak molecular forces in the network overcame the intermolecular interaction and gradually separated from the hydrogel. This process lasts for a period of time, leading to a slow release effect for the loaded drugs. Fig. 4(b) showed that adding different amounts of paeonol to PVA/SF 1/1 hydrogels did not significantly change the mass loss rate of hydrogels; the mass loss rate of paeonol-loaded hydrogels is similar to that of the normal hydrogels, at about 39%.

As shown in Fig. 4(c), for all compositions, hydrogel swelling in water attained equilibrium within 24 h and the swelling ratio at equilibrium decreased as PVA content increased. When PVA/SF was lower than 1/1, the swelling curves of hydrogels showed a gradual upward trend, but when PVA/SF was higher than 1/1, the swelling curves of hydrogels first showed an upward trend and then gradually declined. The reason for this may be that PVA continuously dissolved from the gel during the swelling process and the more PVA content, the more hydrogel dissolved. When the proportion of PVA was relatively high, the amount of PVA dissolved became larger, leading to great impact on the swelling behaviors. The PVA/SF 0.5/1 hydrogel achieved a maximum swelling ratio of 7.36 due to its lower mass loss and stable network in the hydrogel. Fig. 4(d) shows the swelling curves of hydrogels with different paeonol contents when PVA/SF was 1/1. It can be seen that the addition of paeonol has no significant impact on the swelling performance of hydrogels. Because HRP/H_2_O_2_ only react with silk fibroin, the cross-linked network of SF plays a key role in the swelling behavior. Paeonol cannot react with SF or PVA; it only binds with hydrophobic sites from macromonomers. So paeonol has no significant effect on the formation of the hydrogels and the amount of loaded drug was relatively low compared to the components in the hydrogel. Therefore, the addition of paeonol had no significant effect on the swelling rates of hydrogels.

### Structural characterization

**Fig. 5:**
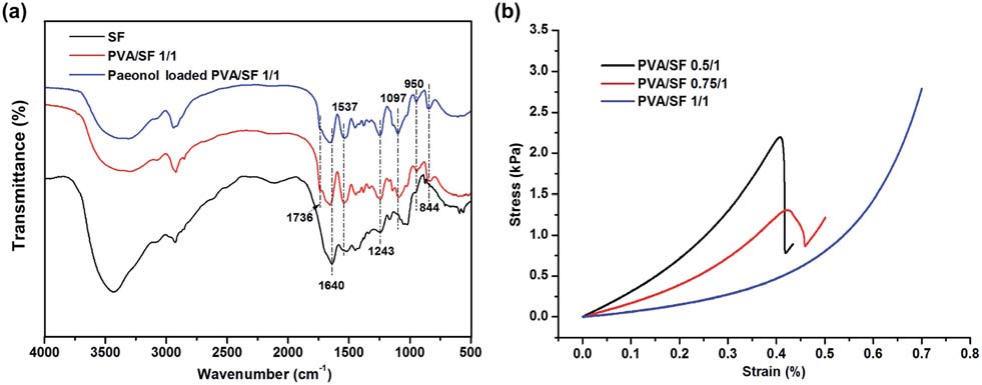
(a) FTIR spectra of SF, PVA/SF 1/1 hydrogels and paeonol-loaded PVA/SF hydrogels. (b) The representative stress–strain curves of different hydrogels.

The FTIR spectra of SF, PVA/SF 1/1 hydrogels and paeonol loaded PVA/SF hydrogels are shown in Fig. 5. Previous literature reported that pure PVA shows absorption bands at 3340, 2942, 1736, 1420–1440, 1326, 1235, 1095, 916, and 850 cm^−1^, respectively attributed to the *ν*(OH), *ν*_a_(CH_2_), *ν*(C=O), *δ*(OH) + *ν*(CH_2_), *δ*(OH) + *γ*_w_(CH), *γ*_w_(CH), *ν*(C–O), *ν*_s_(C–O) and *ν*_r_(C–H) resonances.^28,33^ The absorption bands at 1640 cm^−1^ (amide I), 1537 cm^−1^ (amide II), and 1232 cm^−1^ (amide III) observed in the pure enzymatic crosslinked SF hydrogel curve indicated the coexistence of silk I crystalline structure and random coil conformation.^34^ The PVA/SF 1/1 hydrogel and paeonol-loaded blend hydrogels have similar peaks. The curves are characterized by the presence of absorption bands typical of both components. Moreover, the width of the *ν*(OH) region (3500–3200 cm^−1^) was extended by blending PVA and SF, which could characterize the increase of the intensity of hydrogen bonding. FTIR spectral results implied SF molecules had interactions with PVA.

### Mechanical properties

The stress–strain curves of different gels are shown in Fig. 5(b) and Table 2 summarizes the compression parameters of mechanical testing. From the representational stress–strain curves, all the hydrogels are soft materials which generate high deformation under small force. The compressive modulus *E* of the 8 mm diameter gels declined from 3.012 to 0.592 kPa with the increase of PVA/SF blend ratio up to 1/1. Compressive stress at 30% strain had similar trend. Mechanical properties of gels can be affected by the network density, crosslinking type and specific major skeleton.^35^ In this type of gelation system, fibroin crosslinked by HRP and H_2_O_2_ serves as the main supporting skeleton and thus the mechanical properties of PVA/SF gels are dominated by the fibroin content.

**Table 2:** Compression parameters of blend hydrogels

Sample	Modulus *E* (kPa)	Compressive stress at 30% strain (kPa)
PVA/SF 0.5/1	3.012 ± 0.023	1.300 ± 0.032
PVA/SF 0.75/1	1.652 ± 0.005	0.725 ± 0.013
PVA/SF 1/1	0.592 ± 0.012	0.271 ± 0.024

### Cytotoxicity of the PVA/SF semi-IPN hydrogels

**Fig. 6:**
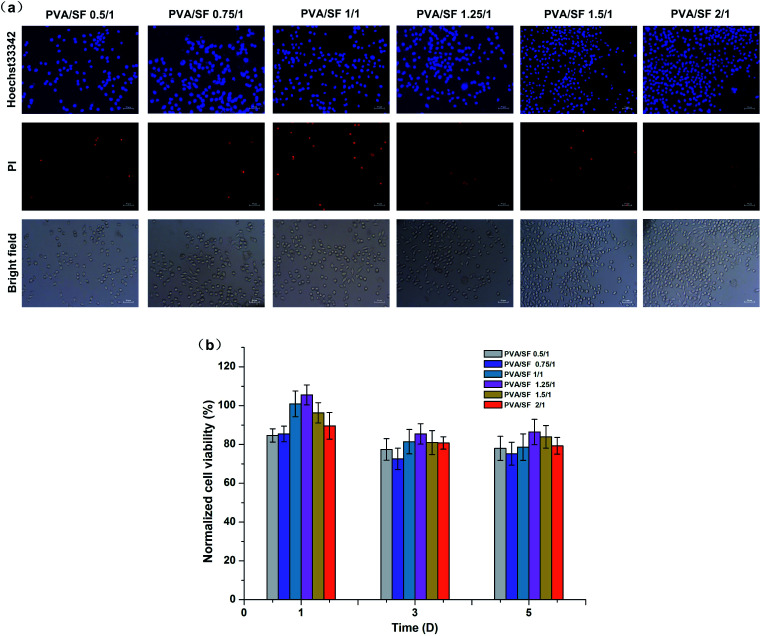
(a) The morphologies of cells growing in the extracted media of different samples after 3 days culture, using 33342 and PI fluorescent dyes. Scale bars, 50 μm. (b) The cell viability assessed by MTT assay after 1, 3 and 5 days exposure to hydrogel extract media.

The extraction method was carried out to compare the cytotoxicity of the different hydrogels. Fig. 6 shows photomicrographs of L929 cells stained with Hoechst 33342 and PI fluorescent dye after 3 days exposure of the cells to extracted media. In this case, Hoechst 33342 and PI staining were used to determine apoptosis cells and dead cells; the apoptotic cells turn to condensed nuclei or fragmented nuclei stained blue and the dead cells were red.^36^ According to Fig. 6(a), there was no significant difference in the number and morphology of cells among experimental groups, except for the PVA/SF 1/1 gel with a slightly higher number of dead cells. Fig. 6(b) shows normalized viability results of L929 cells for 1, 3 and 5 days exposure with the hydrogel extracted media. The normalized viabilities of all the groups were highest on the first day of culture and the maximum value was 105% in the PVA/SF 1.25/1 group. With increase of time, the cell normalized viability of each group decreased slightly, but remained higher than 75%. The cell viability study implied that the hydrogels prepared in this paper were biocompatible, with low cell cytotoxicity. Both SF and PVA have been proven to be non-toxic^18,37^ and enzymatic crosslinking does not introduce toxic reagents, so these hydrogel extracts would also be non-toxic. Therefore, the composite hydrogels can be regarded as safe drug delivery carriers. This study can be further extended to a tissue engineering matrix to develop cell encapsulated hydrogels with mild fabrication conditions, which may help in maintaining cell viability, proliferation and differentiation.

### Paeonol release from the hydrogels

Paeonol was used as the model drug to investigate the release behaviour of hydrophobic drug molecules from PVA/SF 1/1 hydrogels. Because of the limited solubility of paeonol in water, it is difficult to load paeonol by mixing it with pure fibroin solution directly without surfactant and when the paeonol concentration in the mixture is higher than ∼0.2 mg mL^−1^, paeonol will precipitate and distribute ununiformly. However, paeonol was dissolved into PVA solution in advance in this research and then PVA solution containing paeonol was mixed with SF solution, as the emulsifying property of PVA is helpful to improve the solubility of paeonol in aqueous solvent; thus, PVA/SF hydrogels with uniformly distributed paeonol can be obtained. Because PVA is a kind of macromolecular non-ionic surfactant, the hydrophilic group is the alcohol hydroxyl group, which combines with water or SF chains by hydrogen bond, and the hydrophobic group is the long polyethylene chain, which is similar to the hydrophobic substance to be loaded, providing potential binding sites for the drug though hydrophobic effect.^38^ Reducing the interfacial tension between the hydrophobic substance and aqueous matrix is also beneficial for the formation of a homogeneous and stable system matrix with the help of stirring.

**Fig. 7:**
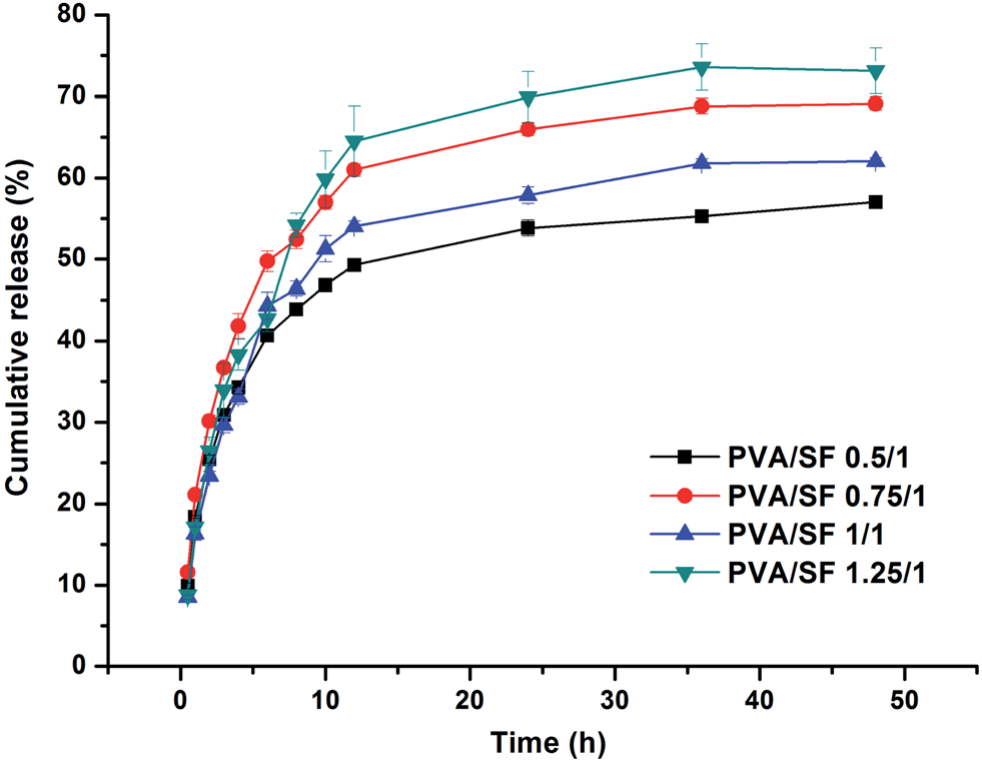
Cumulative release of paeonol from PVA/SF hydrogels over a period of 48 hours.

The percent cumulative release profiles of the paeonol from the hydrogels at 37 °C in PBS over 48 hours are plotted in Fig. 7. There was a continuous incremental rise in the cumulative release of paeonol from the hydrogels. During the first 12 h, the cumulative release rates of paeonol increased rapidly and all the groups exceeded 50% at the end of 12 h. After 12 h, they rose slowly and reached a plateau, with cumulative release rates of paeonol for PVA/SF 0.5/1, PVA/SF 0.75/1, PVA/SF 1/1 and PVA/SF 1.25/1 hydrogels of 57.01%, 69.13%, 62.04% and 73.13%, respectively, at the end of 48 h. The paeonol from the hydrogels exhibited an initial burst release, due to instantaneous release of drug from the surface of the hydrogel. However, after an initial burst release, PVA/SF hydrogel showed slow sustained release of paeonol. This may be due to the PVA simply interpenetrating the SF chains through physical interaction: the paeonol can be released gradually with the slow dissolution of PVA attached to the network by weak molecular force. Also, paeonol is slightly soluble in warm water, so, according to Fick's law of diffusion, paeonol can diffuse from a high concentration in the hydrogel to a low concentration in the release system. In addition, the pore structure, stability and swelling properties of hydrogels simultaneously impact the drug release rates. These results indicate that the PVA/SF hydrogels in different blend ratios with encapsulated drugs can control hydrophobic drug release.

## Conclusion

A series of PVA/SF semi-IPN hydrogels were successfully prepared through the enzymatic crosslinking method. Enzymatic crosslinking effectively shortens PVA/SF hydrogel gelation time. The pore structure of hydrogels can be tuned from oval in shape to lamellar using blend ratios. The stability of PVA/SF hydrogels dropped with the increase of PVA content. Hydrogels swelled to equilibrium within 24 h in water and the swelling ratio at equilibrium decreased as PVA content increased. The compressive mechanical properties of PVA/SF gels are dominated by the fibroin content: the more SF content, the stiffer the hydrogel. Noticeably, hydrogel extracts exhibited biocompatibility, indicating the hydrogels are safe in the fields of drug delivery or tissue engineering. PVA/SF hydrogels with different ratios and encapsulated paeonol achieved the purpose of drug sustained release. Furthermore, the hydrogels were prepared mildly in aqueous media, providing the possibility to load bioactive drugs. The PVA/SF semi-IPN hydrogels obtained in this study are attractive candidates for drug delivery and engineering applications.

## Conflicts of interest

There are no conflicts to declare.

## Supplementary Material
